# Studying Well and Performing Well: A Bayesian Analysis on Team and Individual Rowing Performance in Dual Career Athletes

**DOI:** 10.3389/fpsyg.2020.583409

**Published:** 2020-12-23

**Authors:** Juan Gavala-González, Bruno Martins, Francisco Javier Ponseti, Alexandre Garcia-Mas

**Affiliations:** ^1^Physical Education and Sports Department, University of Seville, Seville, Spain; ^2^Research Group of Physical Activity and Sports, University of Balearic Islands, Palma de Mallorca, Spain

**Keywords:** dual career, social loafing, rowing, team crew, Bayesian analysis

## Abstract

On many occasions, the maximum result of a team does not equate to the total maximum individual effort of each athlete (social loafing). Athletes often combine their sports life with an academic one (Dual Career), prioritizing one over the over in a difficult balancing act. The aim of this research is to examine the existence of social loafing in a group of novice university rowers and the differences that exist according to sex, academic performance, and the kind of sport previously practiced (individual or team). Therefore, a study was conducted from a probabilistic perspective using the Bayesian Network analysis methodology. The results confirm the existence of the Ringelmann effect or social loafing. The Bayesian analysis let us confirm that having a good student who practices a team sport, even in the individual rowing concept, increases the probability of obtaining greater performance (higher number of strokes and more power in each one). Therefore, when rowing partnerships are formed, the occurrence probability chain is quickly simplified, along with values of the top and bottom variables. Finally, the instantiations undertaken on the bottom variable that appears to be common in the two BNs, the watt input, enhance the results obtained. In short, rowers who have a better academic record are more involved in team testing, so this characteristic is defining when it comes to achieving better performance in team testing.

## Introduction

Research on the psychological variables that may affect the performance of sports teams has a long tradition in sports psychology, with key concepts such as sports cohesion (Carron and Brawley, 2000) and cooperation (Garcia-Mas et al., 2006), in addition to recent developments in the synthesis of the currently existing different conceptual frameworks addressed to explain psychological dynamics of the team, e.g., coordination, identification, collective efficacy, or integration (Garcia-Mas et al., 2019). Despite these attempts to clarify the psychological variables responsible for team dynamics, often other basic variables, such as group size, hierarchy, conformity, or social laziness are not considered as relevant either theoretically or practically.

Social loafing is defined as the reduction of individual effort when participating in a group task (Latané et al., 2019). Towards the end of the 19th Century, Maximilien Ringelmann (1913)Ringelmann () studied for the first time the social loafing effect in a tug of war competition. He stated that the maximum result of a team does not equate to the total maximum individual effort (Kosonogov, 2015), with the differences between the real performance and the envisaged performance of the group broadening as the number of members increased. Since then, an extensive line of research has been undertaken on the phenomenon of social loafing, which is now understood as a common phenomenon that occurs in numerous tasks and by both genders (Caracuel et al., 2011; Haugen et al., 2016; Karau and Williams, 2016). In particular, public evaluation or making public the contribution of each member to the total performance of the group, have been highlighted as key situational factors in inhibiting part of social loafing, supporting the idea that this phenomenon could be explained as a loss of motivation caused by a lower degree of recognition or evaluation(Kerr and Bruun, 1983; Harkins, 1987; René et al., 2006; Gavala González, 2010; Gavala-González, 2012; Hagen, 2015; Latané et al., 2019). Furthermore, this effect appears to significantly reduce when the participants feel that they belong to a cohesive group, are satisfied with their role in the team, see high levels of collective performance (Hogg and Vaughan, 2010; Høigaard et al., 2010), are top-level athletes (Jaenes et al., 2018), previously knew the teammates (Kerr and Bruun, 1983; Harkins, 1987) or regularly compete in teams (Czyż et al., 2016). In terms of motivation, when the group atmosphere is perceived as part of the task, social loafing also reduces due to the existence of performance self-referencing rather than the social comparison inherent to an ego-centric atmosphere (Høigaard et al., 2013).

One of the personal variables that has not been studied in depth regarding social loafing is the dual career (DC) of athletes, a social concept that is gradually becoming more relevant in studies on performance athletes. Synthetically, DC is understood to mean the act of combining a sports career and academic studies (Ryba et al., 2015). It is currently very common among all kinds of athletes, regardless of the sport practiced, the country of residence, sex, socio-economic status, and years of experience (Ceciæ Erpiè et al., 2004). According to some authors, it is one of the main challenges faced by top-flight athletes between the ages of 12 and 25, as they have to successfully combine education and high-performance sport (Ryba et al., 2017). Wyllemann and Lavallee (2004) defines four basic stages in athletic careers: initiation, development, mastery, and discontinuation. The Wyllemann and Lavallee model fits into a holistic concept of athletic careers, as it considers the sports dimension together with the other facets that intervene in personal/athletic development, such as the development of personality and self-concept, psychosocial relationships, and academic/vocational aspects, as well as economic and financial aspects.

This model is usually used to understand athletes from a global perspective, which allows critical moments in their DC to be understood in order to prevent situations of conflict and to increase the possibility of living a satisfactory public and private life, maintaining health and wellbeing (Stambulova and Wylleman, 2015). However, we know that it is not always easy to maintain such duality in a healthy way and, as such, pressure and anxiety often arise, along with the abandonment of one of the areas (Gustafsson et al., 2008; Aquilina, 2013; O’Neill et al., 2013; Torregrosa et al., 2015; Sorkkila et al., 2017; Gavala-González et al., 2019).

Following this model, discovering the connection – which has long been studied, – between academic and athletic performance is an area of interest that continues to be a focus of dispute (Holland and Andre, 1987). From a “negative” perspective, some authors have argued that sports practice is an obstacle to academic success (Helsen et al., 1987). In the same vein, other authors (Montecalbo-Ignacio et al., 2017) have tried to show that sport and academic success cannot be achieved at the same time, while only one study found that there was no significant correlation between the academic performance of students and their sporting performance (Acevedo, 2015). Conversely, various studies (Fredricks and Eccles, 2006; Smith et al., 2006; Khan et al., 2016; Deeba et al., 2017; Wretman, 2017) suggest that sport actually improves academic performance for a number of reasons. Among those suggested by authors, is that sports practice reduces leisure time that distracts students, although it is not known if that is solely due to physical practice (individual sports) or to the interaction with a group of people (team sports) (Fox et al., 2010).

Once the area and the conceptual framework that theoretically and empirically underpin it were established, rowing was chosen as the sport of interest for this study on social loafing. Rowing is a mentally and physically challenging sport (Volianitis and Secher, 2009) that requires physical conditioning, endurance, and psychological and physical preparation to compete. Competitions often take place on water, but much of the physical conditioning, training and performance testing takes place on rowing machines (Volianitis and Secher, 2009). These machines accurately simulate the movement pattern and the real rowing situation, falling short only in representing the swaying of the boat and weather conditions.

Regarding the methodology of this study, Bayesian Networks (BNs) are beginning to be widely used in social sciences (Fuster-Parra et al., 2017; Chen and Huang, 2018) and were recently presented as a useful methodology in sports psychology, given their ability to provide information on the probability of occurrence of events related to performance in sports or, for example, the likelihood of sports injuries. BNs also referred to as causal networks or beliefs networks, are a form of statistical modeling which allow us to obtain a graphical network describing the dependencies and conditional independencies from empirical data. The graphical representation of BNs captures the compositional structure of the relations and the general aspects of all probability distributions that factorize according to that structure. They have proven to be a promising tool for discovering relationships between negative features in sport (as is the case of this study; Fuster-Parra et al., 2013), and in many other sport-related studies, such as cooperative teamwork, motivation and types of sporting cooperation among players in competing teams, motivational climate and competitive anxiety, psychological variables related to athlete injuries, and the relative effect of age (Ishigami, 2016; Ponseti et al., 2016a; Olmedilla et al., 2018). Quite recently, a number of papers have been published that use a new approach, namely Dynamic BNs, which strive to predict and then mitigate the probability of injuries occurring in athletes (Peterson and Evans, 2019).

In accordance with the foregoing, the aim of this study is to evaluate the possible existence and its degree – by means of a Bayesian analysis of the Ringelmann effect (or social loafing) in a sample of novice university students rowers. The analysis will include some others variables such as their sex, academic performance, and the kind of sport previously practiced (individual or team).

## Materials and Methods

### Participants

The sample comprised 131 (47 females and 84 males) young adults, all university students, of an average age of 22.36 (SD = 2.01). Regarding the consideration of the participants as Dual-career athletes, the DC global model considers not only the top-level (or elite) athlete-students as a DC persons, but also the athletes of competitive and performance sports, although they are not officially classified as Top Level (Riccardo-Brustio et al., 2020). In this case, we would like to point out that in Spain, the title of High-Level Athletes is granted by the “Consejo Superior de Deportes” (dependent on the Spanish Government) and that of High-Performance Athletes is granted by the Sports departments of the Autonomous Communities (regions). In 2019 (date of the study), there were only 545 people recognized as High Level Athletes, and only 270 athletes were considered High Performance Athletes in the region where the University where the study is located.

The participants of this study were university students and federated athletes who compete at the highest level, they practice daily in their sport discipline and compete at a high level in their sport in leagues and cross-national competitions, but they are rowing novices (without quantifiable experience).

### Ethical Approval

This study was undertaken within the framework of the ERASMUS+ ELIT-in Project and it was approved by the Ethics Committee of the University of Trás-os-Montes and Alto Douro (UTAD), Doc.20/CE/2018 (UTAD 23/2018), which includes the undertaking of the study in line with the Declaration of Helsinki.

### Material and Instruments

In this study, two identical Concept-2 Model D Indoor Rowing Machines were used. This machine replicates on-water training with great efficiency and crossover. It instantly records and stores the following parameters: total test time, partial time every 500 m, meters covered, strokes per minutes, and watt input.

In addition, data corresponding to age, sex, sporting experience in individual and/or team sports, and the academic record of each participant were recorded.

### Experimental Design

The participants were assessed according to a longitudinal strategy in the two different conditions (individual performance and team performance). Several measurements comprising were used: number of meters covered by each rower in three minutes, number of strokes per minutes and the average power exerted (in watts) achieved by each rower in three minutes.

### Procedure

The testing took place in a ventilated and suitable room that the students knew well and often frequented, and where they felt comfortable. The aim was to achieve complete cooperation from the participants, explaining to them the importance of their participation in both tests, which also served to get them as ready as possible for forthcoming competitions.

The rowing machines were placed in the testing room with their display screens covered up so that they had no reference point other than their own perception of effort. Firstly, the participants did their usual warm-up exercises and the procedure was explained to them: A test of 3 min max., first individually (Condition 1) and, after a 24-h rest period, the same warm-up as before and the second condition, as a group in randomly allocated pairs (Condition 2) for the same duration.

In Condition 1, they were individually asked to perform with maximum intensity and separated from the other participants. The instructions were the same for all participants: “You have to try to cover the maximum number of meters possible in three minutes. Please try to give your best performance in this test.”

After a one-day rest period, the time often deemed suitable for total recovery, the “group testing” (Condition 2) was carried out, placing two rowing machines in line formation, replicating the positions in a couple-rower boat. The warm-up and the instructions were the same as in Condition 1, with the exception of informing them that the final evaluated performance would correspond to the total results of both rowers (participants row on their own machines and team score is built with the sum of their individual performance). Although for the study, data were analyzed independiently.

### Data Analysis

As a preliminary step, a descriptive analysis on the variables studied was undertaken, subsequently conducting a t-test to evaluate the differences between them and the eventual statistical significance. The prior verification of the supposed normality of the scores was conducted using the comparative Kolmogorov–Smirnov test. Furthermore, given the nature of the data, a correlation analysis was conducted using Pearson’s R between the performance variables.

The study was undertaken creating BNs, making it necessary to determine the structure of the BN via a Directed Acyclic Graph (DAG) and to assign conditional probabilities to each node of the DAG. The DAG can be obtained only from the data set or using the data set and adding some prior or expert knowledge during the structure learning. Neither expert knowledge nor prior knowledge was used to obtain the model; only our dataset was used. Learning a BN involves the following two tasks: (i) structural learning, in other words, identifying the topology of the BN and (ii) parametric learning, or estimating the numerical parameters (conditional probabilities) given the network’s topology.

Structural learning was used to obtain the BN through the “bn learn package” using R language (Rosselet, 1987). To obtain the structure, the options were to use either a search and score algorithm (Korb and Nicholson, 2010), which assigns a score to each BN structure and selects the model structure with the highest score, or a constraint-based search algorithm (Spirtes et al., 1993), which establishes a conditional independence analysis on the data to generate an undirected graph and convert it into a BN using an additional independence test. The score-based algorithm Tabu (Korb and Nicholson, 2010) was used, which was a plausible model for our data, looking for the structure that best improves the score, e.g., using the highest score.

After building the BN, some instantiations were conducted (injection of hypothetical probabilities) to the bottom variables, as well as observation on how the node and top variables change their probability values. With these instantiations we apply intercausal reasoning to discover the differences in other variables when the bottom variables were artificially changed in their probability. When different causes of the same effect can interact, we called it intercausal reasoning. This type of reasoning allows to observe mutual causes of a common effect and constitutes a very common pattern in human reasoning.

## Results

### Subsection

Table 1 shows the descriptive data of the variables studied in the two research conditions, while Table 2 shows the Pearson correlations between the performance variables in the two conditions. As such, the correlations between the meters covered and the watt input (power) are very high and significant, around 9, but the correlation between the number of strokes in the two conditions (despite also being significant 0.000), is much lower, not reaching the 0.7 value.

**TABLE 1:** Descriptive values of the variables studied.

	**mean**	**SD**	**trimmed**	**Mad**	**min**	**max**	**range**	**skew**	**kurtosis**	**se**
Sex	1.36	0.48	1.32	0	1	2	1	0.58	–1.67	0.04
Individual meters	759.31	88.04	760.58	108.23	482	934	452	–0.21	–0.6	7.69
Individual watts	216.32	71.63	213.97	81.54	54	391	337	0.19	–0.62	6.26
Individual strokes	32.94	4.29	32.95	4.45	23	42	19	0.05	–0.37	0.38
Team meters	742.76	89.51	743.24	106.75	557	980	423	–0.05	–0.85	7.82
Team watts	207.52	72.4	204.3	81.54	83	452	369	0.39	–0.16	6.33
Team strokes	32.39	4.03	32.39	4.45	22	45	23	0.1	0.12	0.35
Age	22.36	2.01	22.3	1.48	18	29	11	0.5	1.11	0.18
Sport type	1.4	0.59	1.45	1.48	0	2	2	–0.4	–0.73	0.05
Academic record	7.77	0.53	7.8	0.43	6.05	8.88	2.83	–0.6	0.32	0.05

**TABLE 2:** Correlations of paired samples (Pearson’s r).

	***N***	***r***	**Sig.**
Individual meters and team meters	131	0.853	0.000
Individual watts and team watts	131	0.867	0.000
Individual strokes and team strokes	131	0.638	0.000

Table 3 shows that there are significant differences between the median values of meters covered and the watt input in both tests (Conditions 1 and 2). That is not the case, however, with the median values in the number of strokes, where no significant differences are observed.

**TABLE 3:** Differences between the median values of the variables studied (*t*-test).

	***X***	**SD**	**Median of standard error**	**95% difference reliability range**		***t***	***gl***	**Sig.(bilateral)**
				**Low**	**High**			
Individual meters – Team meters	16.550	48.159	4.208	8.225	24.874	3.933	130	0.000***
Individual watts – Team watts	8.802	37.125	3.244	2.384	15.219	2.713	130	0.008**
Individual strokes – Team strokes	.550	3.548	0.310	−0.064	1.163	1.773	130	0.079

On verifying the significant differences and the correlation between the median values of the variables, a decision was made to conduct a Bayesian analysis by creating two probability trees corresponding to each of the two separate conditions.

**FIGURE 1:**
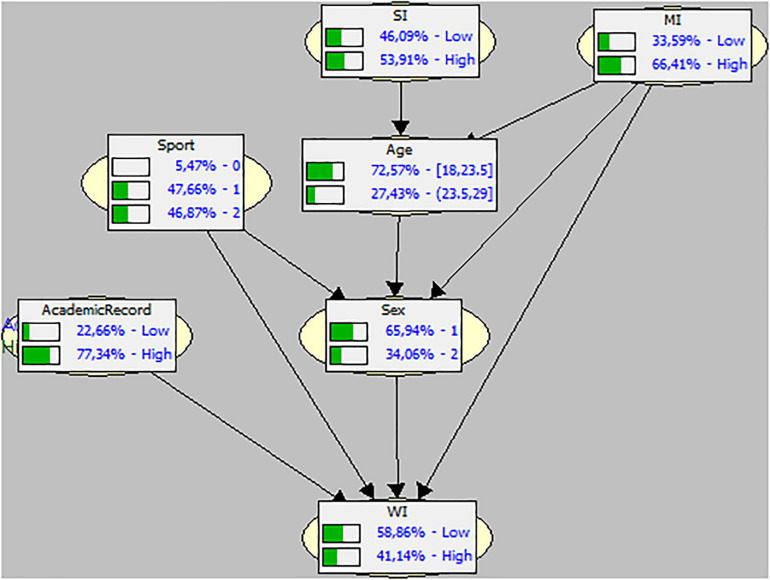
Direct Acyclic Graph (DAG) and Bayesian Network on the studied variables in Condition 1 (individual). MI, meters in individual performance; WI, watts in individual performance; SI, strokes in individual performance.

Figure 1 is a graphic showing the probability values of the BN corresponding to Condition 1 or individual performance. All the variables form one tree. The top or predecessor variables being: the type of sport (at 50% probability between the teams and individuals); the academic record (with a high probability, around 80%); strokes and meters covered. The age and sex are intermediate variables of different probability of occurrence (almost 80%, age, and almost 70%, male). The only bottom variable is the amount of energy exerted by the rower (watts) with a probability distribution of 50% between High and Low.

**FIGURE 2:**
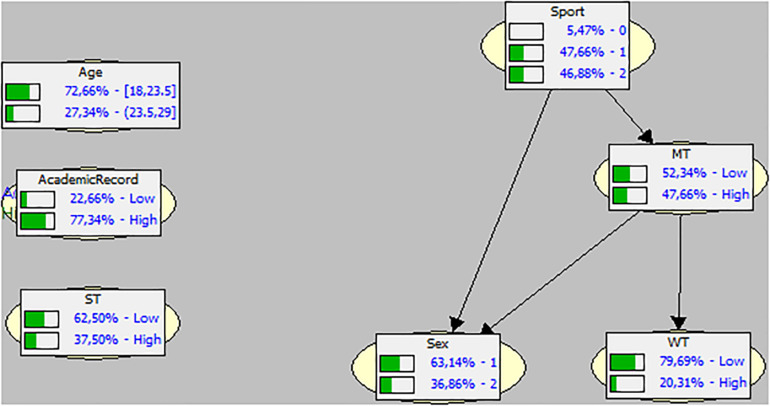
Direct Acyclic Graph (DAG) and Bayesian Network on the studied variables in Condition 2 (team). MT, meters in team performance; WT, watts in team performance; ST: strokes in team performance.

In Figure 2, we can see the DAG graphic and the BN, which are very different both morphologically and in terms of their values with the network corresponding to Condition 1. The tree has lost three variables, which are isolated: the academic record, the number of strokes and the age, which were previously tops and nodes, respectively. The top variable (predecessor) of this tree is the type of sport (with the same probabilistic distribution of the “individual” network). The bottom variables, that is, without a probabilistic impact on the others, are sex (greater probability toward the male sex) and the watts provided by the rowers, which, compared to the individual Condition, is displaced from 60 to 80% of Low probability.

The general picture that we see in the two BN indicates that the probabilities of occurrence of the energy exerted by the rowers are mainly affected by the probability that the rowers have a particular previous history (academic record and type of sport practiced). On the other hand, the condition of practicing a team sport loses probabilistic weight. Thus, we could say, in other words, that the prior learning and background of the athletes studied can be considered as a trigger in the chain of probabilities found.

The BN was validated using a 10-fold cross validation, taking the area under the curve (AUC), accuracy, sensitivity, and sensibility into consideration. Certain terms should first be defined in order to understand the validation used: true positive (TP), true negative (TN), false positive (FP), and false negative (FN). If an observation is labeled correctly within its class, it is considered TP. On the contrary, if an observation is labeled correctly as not belonging to a specific class, it is TN. Both TP and TN suggest a consistent result in the classifier.

However, no classifier is perfect and if the model incorrectly labels an observation as belonging to a certain class, it is considered to be FP; and when incorrectly labeled as not belonging to a certain class, it is designated as FN. Both FP and FN indicate that the results from the classifier are contrary to the actual label (Lalkhen and McCluskey, 2008).

Sensitivity, specificity, and accuracy are described in terms of these concepts: Sensitivity = TP/(TP+FN); Specificity = TN/(TN+FP); and Accuracy = (TN+TP)/(TN+TP+FN+FP).

The AUC shows that the probability of a randomly chosen positive datum being correctly ranked is much higher than for a randomly chosen negative datum (Lalkhen and McCluskey, 2008). The readings provide a complete overview of the performance of the BN. As Tables 4, 5 shows, the validation tables provided some good results (specially in all the individual outcomes), as along with some medium values (mostly in the team Condition). In both conditions, the lesser values correspond with the sport typology, and – in part – with the number of strokes. These validation values should be considered when drawing up both the next step in the Bayesian analysis process (the instantiations) and the final conclusions.

**TABLE 4:** Validations of the variables of the BN in the individual Condition (1).

	**AUC**	**Accuracy**	**Sensitivity**	**Sensibility**
Meters	0.83	0.89	0.90	0.88
Watts	0.83	0.86	0.83	0.89
Strokes	0.59	0.63	0.04	0.81
Age	0.55	0.74	0.96	0.18
Academic record	0.57	0.80	0.19	0.99
Sex	0.87	0.90	0.92	0.87
Sport type	0.59	0.59	0.76	0.42

**TABLE 5:** Validations of the variables of the BN in the team Condition (2).

	**AUC**	**Accuracy**	**Sensitivity**	**Sensibility**
Meters	0.78	0.79	0.87	0.70
Watts	0.51	0.80	1	0
Strokes	0.51	0.61	1	0
Age	0.52	0.70	0.98	0
Academic record	0.51	0.76	0	1
Sex	0.85	0.85	0.81	0.91
Sport type	0.55	0.55	0.83	0.27

### Instantiations

Following the procedure made up in earlier publications C instantiations means the injection of hypothetical vales (usually, the maximum High or Low probability) to some variables according to two conditions: (1) the variables to be instantiated were the ones that turned out to be the bottom variables in the BN and (2) these variables formed part of some of the study’s objectives. In our case, the only variable accomplishing the two conditions is the amount of watts expended in both conditions.

The aim of the instantiations is to outline the changes to be made in the other BN variables, when the instantiated ones where artificially changed in their probability of occurrence. In other words, when a variable is instantiated, the degree of belief about its probability rises towards the variables that are its predecessors and on which it depends probabilistically. The instantiations are used in a complementary way to the BN building, and may provide confirmation of their probability values.

Firstly, there were no prohibited instantiations, but some variables were unaffected by the injection of hypothetical values. Obviously, among them there are those that, in the BN corresponding to Condition 2, were unconnected and others are described in detail. In general, the findings obtained with the instantiations positively parameterize, albeit very selectively, the probability values found in the BN.

In Table 6, we may observe that two variables don’t show any change after the watt (energy produced) instantiations to 100% High in Condition 1 (Individual) was made: sport type and Age. Two more, number of strokes and academic scores shows only very slight changes increasing their High probability of occurrence. The only real and important change appears in the meters variable, increasing its High probability of occurrence in more than 30%.

**TABLE 6:** Instantiation made on the watts produced in Condition 1 (individual) from the obtained values to the maximum High occurrence probabilistic value (100%).

	**Obtained BN**		**Instantiation: 41.14–100% HIGH**	
**Variables**	**High**	**Low**	**High**	**Low**
Academic	77.34	22.66	80.73	19.27
Meters	66.41	33.59	97.26	2.74
Strokes	53.91	46.09	54.05	45.95
**Sex**	**Male**	**Female**	**Male**	**Female**
	65.94	34.60	95.96	4.02
**Age**	**18–24 years**	**24–29 years**	**18–24 years**	**24–29 years**
	72.57	27.43	72.57	27.43
Sport type*	**Individual**	**Team**	**Individual**	**Team**
	47.66	46.88	47.30	48.65

*There is a residual 5.47% without previous sport practice.

The most relevant data that can be seen (Table 7) in the instantiations made to the variable watts (energy produced) both towards 100% High and 100% Low does not cause changes in three variables: academic scores, age and sport types. The remaining two that are affected do so in the same direction, decreasing their probability value when watts is instantiated to 100% Low, and increasing their probability value (High) when instantiating the watts to the maximum value of High (100%). The first one, the number of rowed meters increases by 30% and decreases by more than 10%; while the second, the sex variable, increases 33% (male probability) in High mode, and decreases less than 10% in Low mode.

**TABLE 7:** Instantiation made on the watts produced in Condition 2 (individual) from the obtained values to the maximum High occurrence probabilistic value (100%).

**Variables**	**Obtained BN**			**79.89–100% LOW**			**20.31–100% HIGH**		
	**High**	**Low**		**High**	**Low**		**High**	**Low**	
Academic*	77.34	22.66		77.34	22.66		77.34	22.66	
Meters	47.66	52.34		35.29	64.71		77.34	22.66	
Strokes*		37.50	62.50		37.50	62.50		37.50	62.50
**Sex**	**Male**	**Female**		**Male**	**Female**		**Male**	**Female**	
	63.14	36.86		54.86	54.14		96.76	4.24	
Age*	**18–24 years**	**24–29 years**		**18–24 years**	**24–29 years**		**18–24 years**	**24–29 years**	
	72.66	36.86		72.66	27.34		72.66	27.34	
Sport type**	**Individual**	**Team**		**Individual**	**Team**		**Individual**	**Team**	
	47.66	46.88		47.30	48.65		49.03	38.90	

*In Condition 2 (Team), these variables have no probabilistic impact on others. **There is a residual 5.47% without previous sport practice.

## Discussion

Usually, the majority of the studies conducted are based on the analysis of the connections that arise between different psychological variables. However, there is little tradition of analyzing the connection between one or several of these variables and direct indicators of sports performance (Ponseti et al., 2016b; Núñez-Prats and Garcia-Mas, 2017), or even indirect ones (Leo et al., 2013), which constitute the fundamental objective of this study. In our case, performance was measured through an objective and direct indicator, combining three parameters: number of strokes and meters covered, as well as the watts exerted by each rower.

As regards the results obtained, it should be indicated, pursuant to an extensive tradition of studies on the Ringelmann effect, in our study, through the simulation of the sports discipline of rowing, that the results are congruent with those results found by previous studies (Kugihara, 1999; Caracuel et al., 2011; Haugen et al., 2016; Karau and Williams, 2016) demonstrating clear differences between all the objective performance parameters, as a classical statistical analysis is used, followed by a Bayesian analysis creating two different probability trees, one for each of the two conditions of the study.

However, in the individual rowing condition in the sample studied, the probability values found in our sample clearly indicate that the changes in the probability of the two “epidemiological” tops: academic record (with a really high probability value) and type of sport practiced (individual or team), together with the two behavioral variables, the strokes (at 50% between High and Low) and the meters covered (high probability of occurrence), have an impact on the two nodes and, above all, on the bottom one, which is the value of the watt input or energy exerted (its probability seems to be almost 50% between high and low). The fact that the number of strokes (observable behavior and more easily quantifiable) are not in any case a bottom variable or “daughter” of the other variables, but the watts (the force exerted in each one of them in the two conditions) are, is a very relevant datum that has not previously been observed (Kerr and Bruun, 1983; Caracuel et al., 2011; Høigaard et al., 2013; Czyż et al., 2016; Haugen et al., 2016; Jaenes et al., 2018).

Age, as a node, does not appear to be relevant in terms of its possible impact. In the second tree (Condition 2) the majority of the old nodes are left out of the network, drastically simplifying the chain of probabilities. The academic record, the strokes done and the age, lose probabilistic relevance. But the only predecessor, “father” or trigger variable is the sport practiced by the rowers, the probability of which remains the same as in Condition 1.

The instantiations undertaken on the bottom variable that appears to be common in the two BNs, the watt input, enhance the results obtained. In the individual condition, enhancing the High probability of watt input to 100% does not entail a change in the probability of hardly any variable (including the type of sport), with the exception of the number of meters covered, which increases by a third. This datum poses an interesting aspect, given that no feedback was given through the rowing machine display.

When the same value is set (to 100% High and to 100% Low of watt input, the majority of the variables – in the same line as that shown in the second BN – do not undergo any change in their probabilities. However, the meters covered once again appear to be affected as that observed in the instantiation in the individual condition. Surprisingly, it seems that being a male athlete somehow “prevents” the emergence of the social loafing effect, as when the amount of watts is set to achieve 100% High, more male rowers probability is required, and to achieve a hypothetical 100% Low, that probability has to be inversely reduced.

This datum has not been sufficiently reflected in prior studies on team performance and sex or gender (Kerr and Bruun, 1983; Harkins, 1987; René et al., 2006; Caracuel et al., 2011; Hagen, 2015; Haugen et al., 2016; Karau and Williams, 2016; Latané et al., 2019), nor in those that evaluate the psychological dynamics in team sports (Kerr and Bruun, 1983; Harkins, 1987; Hogg and Vaughan, 2010; Høigaard et al., 2010, 2013; Czyż et al., 2016; Jaenes et al., 2018).

Added to that, in the individual rowing condition, the top variables, that is, those whose probability of occurrence have a cascade effect on the other variables, are the academic record – the higher the probability that the rower is a good student and practices a team sport, even in the individual rowing condition, the higher the probability will be of giving a better performance, which is quantifiable in the higher number of strokes and, above all, in the exertion of force in each one.

The impact on these data on the concept of dual academic career is evident, focusing on an aspect not previously commonly observed. No precedent has been found neither in the prior studies on DCs (Ceciæ Erpiè et al., 2004; Wyllemann and Lavallee, 2004; Gustafsson et al., 2008; Aquilina, 2013; O’Neill et al., 2013; Stambulova and Wylleman, 2015; Stambulova et al., 2015; Torregrosa et al., 2015; Ryba et al., 2017, 2015; Sorkkila et al., 2017; Gavala-González et al., 2019), nor in the majority of those that focus on the link between academic and sporting performance (Helsen et al., 1987; Holland and Andre, 1987; Acevedo, 2015; Montecalbo-Ignacio et al., 2017).

The results can be considered from a different point of view too: academic performance may be a contributing factor to sports performance, beyond its well-studied role as a relevant psychosocial factor. In this strict sense, it has not been possible to find direct precedents about it in the extensive existing literature on the subject (Ceciæ Erpiè et al., 2004; Wyllemann and Lavallee, 2004; Gustafsson et al., 2008; Aquilina, 2013; O’Neill et al., 2013; Stambulova and Wylleman, 2015; Stambulova et al., 2015; Torregrosa et al., 2015; Ryba et al., 2017, 2015; Sorkkila et al., 2017; Gavala-González et al., 2019), nor in the majority of those that focus on the link between academic and sporting performance (Helsen et al., 1987; Holland and Andre, 1987; Acevedo, 2015; Montecalbo-Ignacio et al., 2017). The only finds obtained in that sense (Fredricks and Eccles, 2006; Smith et al., 2006; Khan et al., 2016; Deeba et al., 2017; Wretman, 2017) attribute this link it to the fact that sports practice or the interaction with a group of people (team) reduces their leisure time, which increases the probability of them focusing more intensely on the activity (sport or studies) that they are doing at the time (Fox et al., 2010).

## Conclusion

Firstly, we cannot speak of only one objective performance indicator, as it has been demonstrated that the strokes done, meters covered and watts contributed do not behave the same with regard to the probabilities of occurrence. This fact may reaffirm the existence of the Ringelmann effect, as, in team rowing, the rhythm of the strokes is usually maintained due the constant synchronization of the rowers (equal number of strokes), but under no circumstance does this mean that they contribute the same watts of energy in each stroke. Furthermore, an external observer (coach, spectator and even in the usual video feedback) do not perceive differences in the effort of rowers, even though they exist.

Secondly, in the individual rowing condition, the top variables, that is, those whose probability of occurrence have a cascade effect on the other variables, are the academic record – the higher the probability that the rower is a good student and practices a team sport, even in the individual rowing condition, the higher the probability will be of giving a better performance, which is quantifiable in the higher number of strokes and, above all, in the exertion of force in each one.

The strictly behavioral variables lose their probabilistic impact capacity and the experience of being a member of a sports team only becomes a predecessor or trigger variable and, as a sole bottom variable – that is, that which is affected by the probabilities of occurrence of all the other variables, is the variable of watt input or energy and effort exerted by the rowers in the strokes, confirming that observed in the individual condition.

Finally, the instantiations undertaken on the bottom variable that appears to be common in the two BNs, the watt input, enhance the results obtained. In the individual condition, enhancing the High probability of watt input to 100% does not entail a change in the probability of hardly any variable (including the type of sport), with the exception of the number of meters covered, which increases by a third. This datum poses an interesting aspect, given that no feedback was given through the rowing machine display, in the same line as the proprioceptive feedback that appears in the BN of the team condition: could it be that there is an internal perception of the meters covered, which in some way substitutes – completely or to a large extent – knowledge of the standard results, in rowers with more experience and who are trained to mentally calculate the number of strokes done and meters covered with each one? Further research will have to be conducted on this line of study.

When the same value is set (to 100% High and to 100% Low of watt input, the majority of the variables – in the same line as that shown in the second BN, do not undergo any change in their probabilities. However, the meters covered once again appear to be affected – in the same sense, both in terms of increase and decrease – as that observed in the instantiation in the individual condition. Surprisingly, it seems that being a male athlete somehow “prevents” the emergence of the social loafing effect, as when the amount of watts is set to achieve 100% High, more male rowers probability is required, and to achieve a hypothetical 100% Low, that probability has to be inversely reduced.

This datum has not been sufficiently reflected in prior studies on team performance and sex or gender (Kerr and Bruun, 1983; Harkins, 1987; René et al., 2006; Caracuel et al., 2011; Hagen, 2015; Haugen et al., 2016; Karau and Williams, 2016; Latané et al., 2019), nor in those that evaluate the psychological dynamics in team sports (Kerr and Bruun, 1983; Harkins, 1987; Hogg and Vaughan, 2010; Høigaard et al., 2010, 2013; Czyż et al., 2016; Jaenes et al., 2018). Given its practical implications, it deserves to be thoroughly studied in future research.

Finally, it has appeared that the influence of the sex of the rowers is relevant for the effectiveness of the team, altering the effect of social laziness.

## Limitations of This Study

There was a relevant methodological limitation that corresponds to the low number of variables studied, which clearly affected their validity on completing the BN. This fact has been reflected in the scope and generalization of discussion on the results and conclusions. This is mainly due to the characteristics of the Bayesian analysis that, even though the missing variables are suitably supported, are affected by the low number of variables included in the study or, conversely, by their high number (Scanagatta et al., 2018).

Secondly, the use of instantiations through the “injection” of hypothetical data, was cautiously considered when forming conclusions and, above all, the practical consequences of them.

## Practical Applications and Future Developments

The data obtained in this study produce some practical effects. It must be taken into account that the competition rowers are selected from laboratory tests, so the biomechanical control of the stroke movement, or the use of some feedback criteria different to that used to date, should be improved.

In addition, considering that no attention is usually paid to the condition of the rowers’ team, it is possible to think about the prevention of social loafing during the formation period, and in the competitive stage. With raining rowers on team building, or similar techniques, that increases the strength of the psychological team dynamics, particularly in terms of coordination, cohesion, and cooperation (Bruner and Spink, 2010; Leo et al., 2013; Olmedilla et al., 2018), they can gain weight in the design of psychological-technical approaches aimed at improving crew performance with more than one rower.

Although it is already seriously considered at present, the protection of the academic career of not only elite athletes, but also those of performance, should be extreme, given the positive synergy observed.

Finally, regarding the data obtained, could it be that there is an internal perception of the meters covered, which in some way substitutes –completely or to a large extent – knowledge of the standard results, in rowers with more experience and who are trained to mentally calculate the number of strokes done and meters covered with each one? Further research will have to be conducted on this line of study.

## Data Availability Statement

The raw data supporting the conclusions of this article will be made available by the authors, without undue reservation.

## Ethics Statement

This study was undertaken within the framework of the ERASMUSCC ELIT-in Project and it was approved by the Ethics Committee of the University of Trás-os-Montes and Alto Douro (UTAD), Doc.20/CE/2018 (UTAD 23/2018), which includes the undertaking of the study in line with the Declaration of Helsinki. The patients/participants provided their written informed consent to participate in this study.

## Author Contributions

JG-G. and AG-M: conceptualization, investigation, writing—review and editing, writing—original draft preparation, and project administration. JG-G, FP, and AG-M: methodology. BM and FP: software, validation, data curation, and visualization. BM, FP, and AG-M: formal analysis. JG-G, BM, FP, and AG-M: resources. AG-M: supervision. FP and AG-M: funding acquisition. All authors have read and agreed to the published version of the manuscript.

## Conflict of Interest

The authors declare that the research was conducted in the absence of any commercial or financial relationships that could be construed as a potential conflict of interest.
